# Modified poly(methyl methacrylate) bone cement in the treatment of Kümmell disease

**DOI:** 10.1093/rb/rbaa051

**Published:** 2021-01-09

**Authors:** Jinjin Zhu, Shuhui Yang, Yute Yang, Teng Yao, Gang Liu, Shunwu Fan, He Zhao, Fuzhai Cui, Xiumei Wang, Guoqiang Jiang, Xiangqian Fang

**Affiliations:** 1 Department of Orthopaedic Surgery, Sir Run Run Shaw Hospital, Zhejiang University School of Medicine, Hangzhou 310016, China; 2 Key Laboratory of Musculoskeletal System Degeneration and Regeneration Translational Research of Zhejiang Province, Hangzhou 310016, China; 3 State Key Laboratory of New Ceramics and Fine Processing, School of Materials Science and Engineering, Tsinghua University, Beijing 100084, China; 4 Department of Spinal Surgery, The Affiliated Hospital of Medical School of Ningbo University, Ningbo 315020, China; 5 Department of Orthopedics, Dongzhimen Hospital, Beijing University of Chinese Medicine, Beijing 100700, China

**Keywords:** mineralized collagen, poly(methyl methacrylate) bone cement, Kümmell disease, mechanical property

## Abstract

Kümmell disease (KD) causes serious vertebral body collapse in patients. However, only a few case reports have been conducted and the number of patients with KD investigated was limited. Additionally, the frequently used poly(methyl methacrylate) (PMMA) bone cement for KD is limited by excessive modulus and poor biocompatibility. Herein, we aimed to modify PMMA bone cement with mineralized collagen (MC), and compare the clinical effects, image performance and finite element analysis between the modified bone cement and PMMA bone cement for the treatment of phase I and II KD. Thirty-nine KD patients treated with PMMA bone cement and 40 KD patients treated with MC-modified PMMA bone cement from June 2015 to March 2017 were retrospectively analyzed. The surgical procedure, intraoperative blood loss, hospital stay and complications were compared between different groups. Visual analog scale, Oswestry disability index, anterior vertebral height, posterior vertebral height, computed tomography value, adjacent vertebral re-fracture, Cobb angle and wedge-shaped correction angle were evaluated. Additionally, the representative sample was selected for finite element analysis. We found that the MC-modified PMMA bone cement could achieve the same effect as that of PMMA bone cement and was associated with better vertebral height restoration in the long term.

## Introduction

Patients with Kümmell disease (KD) are initially asymptomatic for weeks to months. Thereafter, the patients may experience vertebral body collapse after a minor trauma, recurring pain at the same site aggravates and the disease progresses to kyphosis, thereby leading to a series of complications [1]. However, only a few case reports have been conducted and the number of patients with KD investigated was limited. Currently, several targeted therapeutic methods, such as surgical intervention and conservative treatments, including analgesics and bed rest, have been proposed. To achieve postoperative pain relief and early ambulation [2] in patients with KD with a minor trauma, percutaneous vertebroplasty (PVP) using poly(methyl methacrylate) (PMMA) bone cement is widely employed for the treatment [3−6].

However, PMMA bone cement lacks osseointegration ability and has a higher elastic modulus, which could easily lead to loosening and displacement of bone cement, cause postoperative adjacent vertebra fractures and re-compression fractures, thereby requiring further surgical intervention [7, 8]. The incidence of new fractures has been reported to be 5.0–22.0% and 17.2–52.0% in 1 and 4 years postoperatively, respectively [9, 10]. Moreover, PMMA bone cement could easily leak because of its fluidity and the osteonecrosis in patients with KD, which could in turn result in cardiopulmonary embolism and other complications [2]. In addition, the integration between the bone cement and tissue is inadequate, and the incidence of complications, such as vertebral height reduction, is high [11].

Mineralized collagen (MC) is mainly composed of type I collagen, which is derived from cattle Achilles tendon, and hydroxyapatite (HA). Through biomineralization technology, the chemical and physical properties of the functional composite materials of MC met the requirements of the human internal environment. In our previous study, we modified the PMMA bone cement by adding MC [12−16]. We performed *in vitro* and *in vivo* studies of MC-modified PMMA bone cement and evaluated its improvement in relation to the biological and biomechanical shortcomings of PMMA bone cement alone [2, 17]. However, the MC-modified PMMA bone cement has not been evaluated in the treatment of KD, which has suitable modulus and bioactivity for bone regeneration. Hence, in this study, we applied the MC-modified PMMA bone cement for the treatment of KD. This is the first clinical application of MC-modified PMMA bone cement in KD. We compared the visual analog scale, Oswestry disability index (ODI), anterior vertebral height (AVH), posterior vertebral height (PVH), computed tomography (CT) value, adjacent vertebral re-fracture, Cobb angle and wedge-shaped correction angle in the two groups. Additionally, we investigated the possible mechanism of the improvement in the mechanical properties of the bone cement with MC via finite element analysis.

## Materials and methods

### Surgical procedures and materials

This study was approved by the Affiliated Hospital of the Medical School of Ningbo University. Patients with osteoporosis (*T* value of bone mineral density (BMD) <3.0) were included, and patients with spinal trauma, deformities, tumors, and previous spinal surgery history and those who were pregnant were excluded. Before the surgery, CT scans of the thoracolumbar spine were performed, and the CT scan data were also obtained after the examinations.

The basic operation was the same as that previously reported [2]. The puncture route was clearly defined by a puncture needle in the coronal plane: upper quadrant of bilateral pedicles. The angle of the needle to the bilateral pedicle was ∼12°−18° in the horizontal plane. MC powder was prepared as previously described [12]. Phosphate and soluble calcium were added to an acidic collagen solution. Briefly, sodium hydroxide solution was slowly added dropwise to increase the pH level, which caused the calcium and phosphate ions to nucleate and subsequently grow into nano-HA along the collagen fibers (i.e. MC). When the pH of the mixture approached neutral, MC was precipitated. PMMA bone cement (Mendec Spine Cement; Tecres SPA, Verona, Italy) is commercially available in PVP. The MC-modified PMMA bone cement was prepared by mixing the MC particles with PMMA powder, which was followed by the addition of liquid MMA to the PMMA and MC powder mixture. The optimal mix ratio of MC particles was 15% by weight.

### Patient information

**Figure 1.**
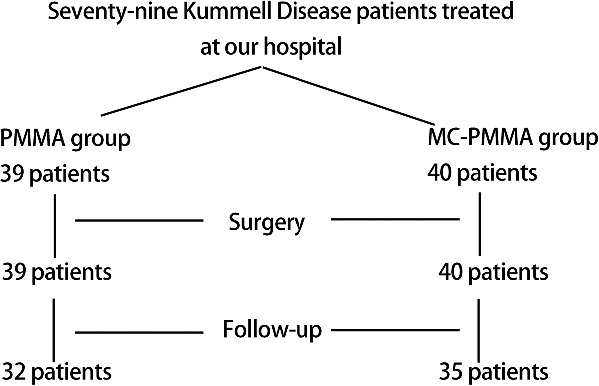
Follow-up scheme. PMMA group (treated with PMMA bone cement; *n* = 39) and MC-modified PMMA group (treated with MC-modified PMMA bone cement; *n* = 40), respectively. Thirty-two patients in the PMMA group and 35 patients in the MC-modified PMMA groups were followed up for 1 year

Thirty-nine patients with KD (13 male and 26 female; average age 71.54 ± 7.87 years) treated with PMMA bone cement and forty patients with KD (12 male and 28 female patients; average age 71.19 ± 11.21 years) treated with MC-modified PMMA bone cement from June 2017 to March 2018. The patients with KD were assigned randomly to either the PMMA group or MC-modified PMMA group using permuted block randomization. The specific follow-up scheme is shown in Fig. 1. According to Li's [18] classification, we classified KD as type I (intact or mild wedge in the anterior column), type II (intact posterior cortical wall with >20% anterior body height loss) and type III (severe collapse with posterior cortical loss). The PMMA group has 13 type I KD and 26 type II KD cases, and the MC-modified PMMA group has 13 type I KD and 27 type II KD cases (Table 1).

**Table 1.** Patients’ clinical data and observation index ($\overset{-}{\chi }\pm s)$

Parameters	MC-PMMA group	PMMA group
Number of patients	40	39
Mean age (years)	71.19 ± 11.21	71.54 ± 7.87
Gender (male/female)	12/28	13/26
Staging of disease(I/II )	13/27	12/27
Injectable time（min）	8.8 ± 0.5	9.7 ± 0.7
Injected amount（ml）	4.3 ± 1.1	4.2 ± 1.2
Incidence of adjacent vertebra re-fracture (%)	10.00%^**^	38.46%
Leakage rate (%)	10.00%	23.08%
Amount of bleeding (ml)	13.3 ± 3.5	14.3 ± 3.3

** Significant difference between the corresponding groups (as indicated by the horizontal bar) at *P* < 0.01.

* Significant difference between the corresponding groups (as indicated by the horizontal bar) at *P* < 0.05.

All patients satisfied the following inclusion criteria: (i) no or only minor history of trauma; (ii) progressively aggravated axial back pain; (iii) spinal fixation tenderness with varying degrees of movement disorders; (iv) delayed vertebral compression based on imaging and presence of vacuum fissures in the vertebral body based on imaging; (v) hydrops sign in the liquid-containing fissure area based on magnetic resonance imaging (MRI) (i.e. low signal on T1 imaging and high signal on T2 imaging), low signal on T1 and T2 images in gaseous fissure area, and uneven signal in gas−liquid mixture. Bone resorption and/or sclerotic bone formation was present in the lesions. Moreover, X-ray, MRI and CT examinations were performed to exclude pain caused by compression of the dural sac and nerve root due to space-occupying lesions, intervertebral disc lesions and spinal stenosis. No swelling of the lower half and lower endplate of the vertebral body was found.

### Observation indicators

Operation time, intraoperative blood loss, duration of hospital stay of each patient and the amount of intraoperative bone cement injection were recorded. Visual analogue scale (VAS) and ODI scores before operation, 3 days after operation, and at each follow-up were used to evaluate the extent of pain relief. AVH, PVH and CT value (indicating the density of a local tissue or organ [14]) of the injured vertebra were measured, and the average was calculated before operation, 3 days after operation and at each follow-up. The incidence of postoperative complications was statistically analyzed, and the correction rates of the Cobb angle and wedge-shaped angle of the injured vertebrae and the adjacent vertebral re-fracture, leakage and complication rates were calculated by CT.

### Finite element analysis sample acquisition

The spine of a 58-year-old healthy adult female volunteer was scanned with a 320-slice spiral CT (Aquilionone TSX.301A, Toshiba, Japan) after informed consent was obtained. The CT scan data were stored in DICOM format, and imported into Materialise’s Interactive Medical Image Control System (MIMICS), and Mimics Research 17.0 Software (Materialise Mimics, Belgium) was used for preprocessing. For 3D reconstruction, the Tl2-L2 vertebral bone was separated and selected from the background image using the region growing tool, according to the CT value. Each articular facet joint was segmented manually to form three independent masks. After manual repair and filling, 3D models were generated. The digital forward engineering software 3-matic 9.0 (Materialise Mimics) was used to guide further smoothing and repairing. Moreover, the model was segmented into cortical bone, cancellous bone, endplate, fibrous ring and nucleus pulposus by Boolean operation.

### Vertebra models with and without KD

Because fractures of KD are most common in L1, the defect model simulating KD was established via removing part of the cortical bone and cancellous bone of the L1 vertebral body. All model parts were converted into 3D solid models after fast paving and stored in Initial Graphics Exchange Specification (IGES) format. All IGES files were guided by the general finite element software Ansys Workbench 17.0 DM module (Ansys Company, USA), and the spine model was assembled. A sphere with a volume of 6 cm^3^ was formed in the cancellous bone of the L1 vertebral body center to simulate the injection of bone cement, and a cylindrical-like body with a radius of 2 mm was generated. The Ansys automated mesh generation function was used to network the model lattice division.

### Material properties and contact types

Based on previous literature on KD, the material properties of the models of cortical bone, cancellous bone, endplate, fibrous ring, nucleus pulposus and bone cement were provided using the assembly model import module [19]. The contact types were defined as follows: frictional mode with a friction coefficient of 0.2; bonded mode with no separation between the vertebral body and intervertebral disc; and bonded mode with cement and reinforced vertebral body [20] (Tables 2 and 3).

**Table 2.** Material properties

Material	Modulus of elasticity (MPa)	Poisson ratio	Thickness (mm)	Density (kg/m^3^)
PMMA	1600.00	0.33	-	-
MC-PMMA	1132.00	0.32	-	-
Cortical bone	8040.00	0.30	1.00	2000.00
Cancellous bone	34.00	0.32	-	1100.00
Cartilaginous endplate	670.00	0.40	0.60	2000.00
				
Fibrous ring	2.56	0.45	-	1200.00
Nucleus pulposus	1.00	0.50	-	1200.00

**Table 3.** Ligament properties

Ligament	Modulus of elasticity (MPa)	Poisson ratio
Anterior longitudinal ligament	7.8	0.3
Posterior longitudinal ligament	10.0	0.3
Ligamentum flavum	17.0	0.3
Intertransverse ligament	10.0	0.3
Arthrocapsular ligament	7.5	0.3
Interspinous process ligament	10.0	0.3
Supraspinous ligament	8.0	0.3

### Boundary conditions and loads

All models were fixed at the bottom of the L2 vertebral body (six-way displacement was 0). A 500-N vertical downward force was applied to the upper surface of the T12 vertebral body as a preload to simulate the upright condition of the human thoracolumbar spine. The pure moments of forward bending, backward extension, left bending, right bending, left-handed rotation and right-handed rotation were applied on the upper surface of the T12 vertebral body, with an amplitude of 15 N·m [21], simulating the human body’s load-bearing and bending conditions in different directions. A vertical force of 500, 700, and 1000 N was used to simulate the human body’s load [22].

### Model validation

The degree of freedom of motion of a single segment of the complete spinal model established in this study was compared to the classical *in vitro* thoracolumbar biomechanical test results of Schuhz *et al*. [23]. In Schuhz’s biomechanics study, the verification conditions were established under a 400-N vertical preload. All directions of forward bending, backward extension, left bending, right bending and clockwise rotation were applied to the upper vertebral body of a single intervertebral disc of the model, with magnitudes of 4.7 and 10.6 N·m. The means and standard deviations of a single segment activity were compared [22].

### Statistical analysis

Data were expressed as mean ± SD and analyzed by SPSS 13.0 software (SPSS software 13.0, Chicago, IL, USA). Student’s *t*-test was used to compare quantitative variables pre- and postoperatively and $\chi^{2}$ test to compare the incidence of postoperative complications. A *P* values <0.05 was considered statistically significant.

## Results

The systematic follow-up scheme is shown in Fig. 1. All 79 patients were followed for 4–16 months, and 67 patients completed at least 1-year of follow-up. The MC-modified PMMA group had almost the same operational performance (injection time and amount) as that of the PMMA group. The operation time of the MC-modified PMMA and PMMA groups was 27.4 ± 3.4 and 26.0 ± 4.2 min, respectively. The amount of MC-modified PMMA bone cement introduced was 4.3 ± 1.1 ml and that of PMMA bone cement was 4.2 ± 1.2 ml. Mean age, amount of bleeding, hospital stay and BMD of the 79 patients of two groups were almost similar (Table 1). Mean age of the MC-modified PMMA group was 71.2 ± 11.2 while that of the PMMA group was 71.5 ± 7.9. The amount of bleeding of the MC-modified PMMA group was 13.3 ± 3.5 ml and that of the PMMA group was 14.3 ± 3.3 ml. The hospital stay of the MC-modified PMMA group was 7.5 ± 1.6 days and that of the PMMA group was 7.4 ± 1.7 days. The BMD value based on CT was 2.9 ± 1.0 for the MC-modified PMMA group and 2.9 ± 1.3 for the PMMA group.

**Figure 2.**
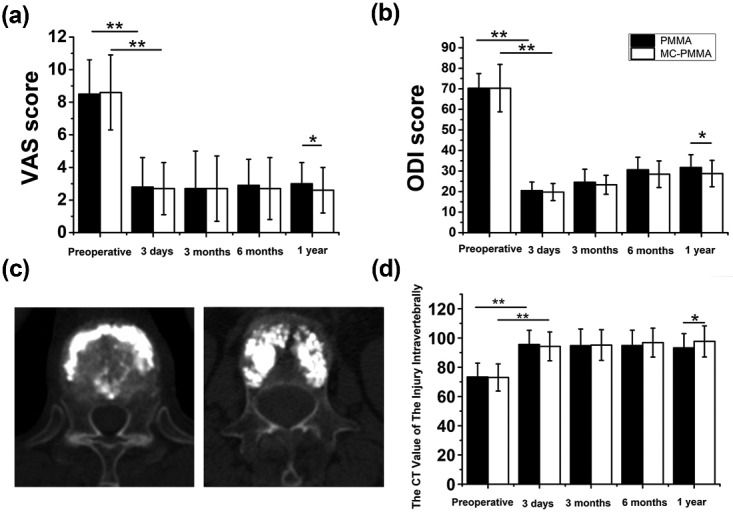
VAS, ODI, CT Value and cross-sectional view. (**A**) VAS scores and ODI scores: VAS and ODI scores were evaluated and compared between the MC-modified PMMA group and PMMA group preoperatively and at 3 days, 3, 6 months, and 1 year postoperatively. (**B**) CT value and cross-sectional view: cross-sectional view of the injured vertebra in the MC-modified PMMA group at 1-year follow-up. The modified bone cement was stable in the vertebral body and fused with the surrounding tissues, which tended to become smaller; the number of surrounding trabeculae continued to increase significantly. Cross-sectional view of the injured vertebra in the PMMA group at 1-year follow-up. The interface between the PMMA bone cement and surrounding tissues is clear. The CT value differed between the MC-modified PMMA and PMMA groups at 1-year follow-up

The MC-modified PMMA group had a lower cement leakage rate (10.00%) than the PMMA group (23.08%) and has a lower incidence of adjacent vertebral re-fracture (*n* = 15) than the PMMA group (*n* = 4) (*P* < 0.05). No cement was released from the posterior edge of the vertebral body into the spinal canal, and no clinical symptoms due to cement leakage were noted in both groups (Table 1). VAS and ODI scores at 3 days, 3 and 6 months after surgery significantly improved in both groups (∼8.5 and 70, respectively; *P* < 0.05). However, no significant difference between the two groups was found (*P* > 0.05) except at 1-year follow-up (*P* < 0.05) (Fig. 2A and B). In addition, the CT value in the MC-modified PMMA group was significantly higher than that in the PMMA group at 1-year follow-up (*P* < 0.05). The CT value of the injured vertebra in the MC-modified PMMA group 3 days postoperatively was significantly higher than that preoperatively (*P* < 0.05); similarly, the CT value of the injured vertebra in the PMMA group 3 days postoperatively was higher than that before surgery; however, no statistically significant difference was observed (*P* > 0.05) (Fig. 2C and D).

**Figure 3.**
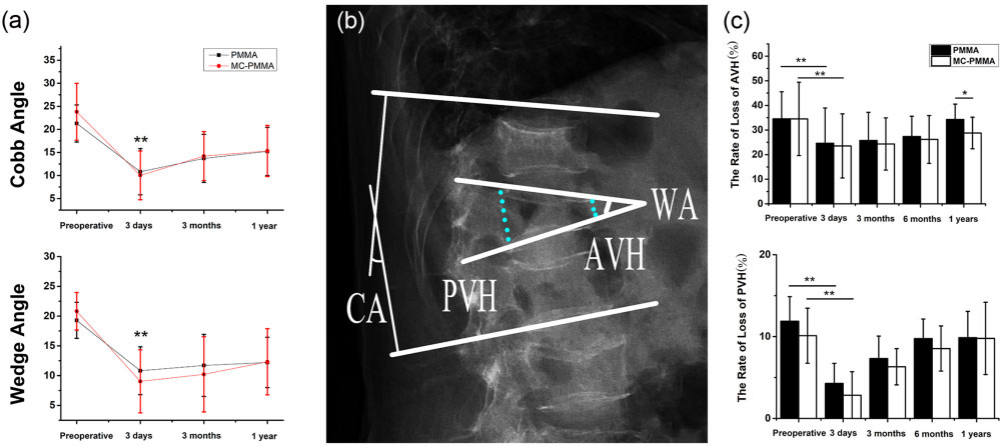
Comprehensive measurement. (**A**) The Wedge angle and the Cobb angle in PMMA and MC-modified PMMA groups. (**B**) The measurement method of kyphotic angle (Cobb angle, CA), correction rate of the wedge-shaped angle of the injured vertebrae (WA), the AVH and PVH. (**C**) AVH and PVH data. The rate of loss of AVH = (the mean height of the anterior border of the injured vertebrae − the height of the leading edge of the injured vertebra)/the mean height of the leading edge of the upper and lower vertebral bodies of the injured vertebra × 100%. The rate of loss of PVH = (the mean height of the posterior border of the injured vertebrae − the height of the posterior margin of the injured vertebrae)/the mean height of the posterior border of the upper and lower vertebral bodies of the injured vertebrae × 100%. If the body has a wedge or flat vertebra, the normal vertebral body closest to the injured vertebra is used for the measurement

The Cobb and wedge angle between the two groups decreased significantly after the surgery and were almost the same with a slight increase at 1-year follow-up (Fig. 3A). The height of the injured vertebral body at each follow-up significantly improved in both two groups (*P* < 0.05), especially AVH. The rate of loss of AVH in the PMMA group was from 0.35 ± 0.13 to 0.25 ± 0.16 and that in the MC-modified PMMA group was from 0.36 ± 0.14 to 0.23 ± 0.17. The rate of loss of PVH in the PMMA group was from 0.12 ± 0.07 to 0.04 ± 0.06 and that in the MC-modified PMMA group was from 0.10 ± 0.08 to 0.03 ± 0.05. However, the height of the injured vertebral body was undetermined with prolongation of the follow-up period in both groups, especially in the PMMA group; nevertheless, a significant difference between the two groups (*P* < 0.05) at 1-year follow-up was found, especially in AVH. The rate of loss of AVH in the PMMA group was 0.34 ± 0.16 while that in the MC-modified PMMA group was 0.29 ± 0.13.

**Figure 4.**
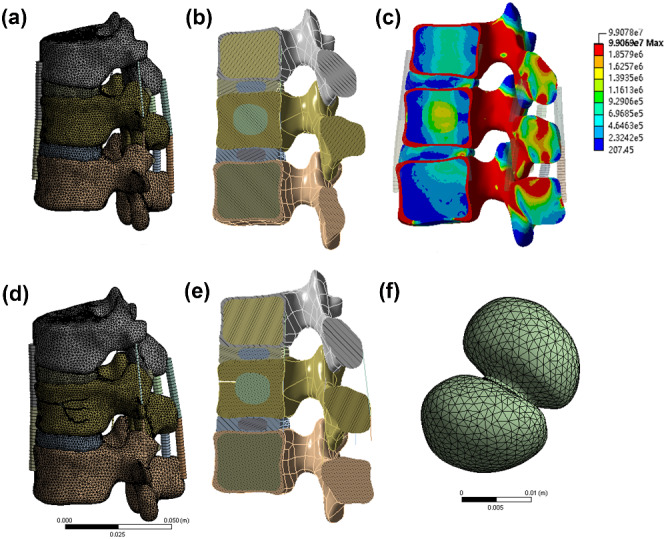
Vertebral body model. (**A**) Normal model of a human vertebra. (**B**) Internal structure of vertebral body. (**C**) Stress distribution of vertebral body. (**D**) Fracture model of a human vertebra. (**E**) Internal structure of vertebral body. (**F**) PMMA bone cement with and without MC model

**Figure 5.**
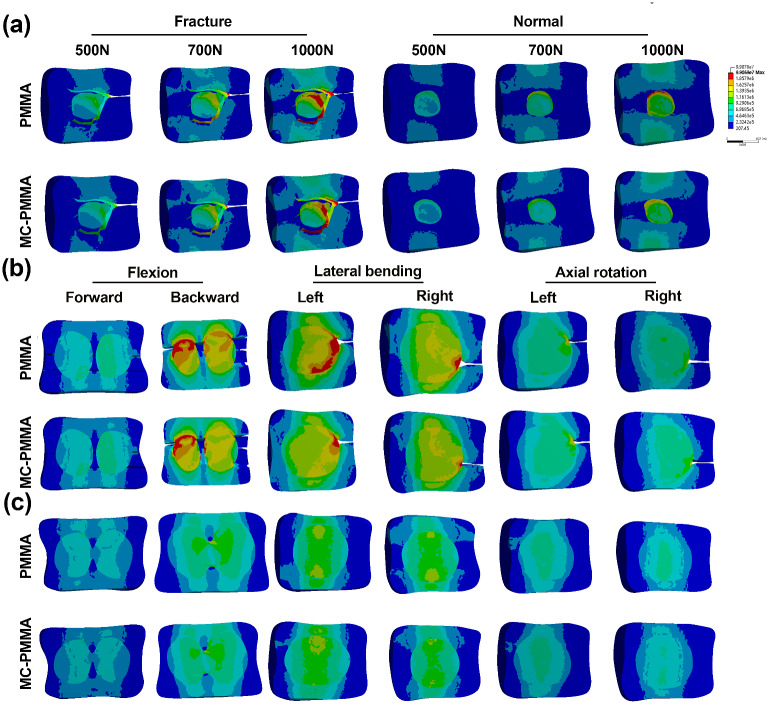
Vertebral body stress. (**A**) Stress nephogram of bone cement periphery of fracture and normal models under 500, 700 and 1000 N. (**B**) Stress nephogram of bone cement periphery of fracture model under forward and backward flexion, left and right lateral bending, left and right axial rotation. (**C**) Stress nephogram of bone cement periphery of normal model under forward and backward flexion, left and right lateral bending, left and right axial rotation

Figure 4 shows the constructed Kümmell and normal models of human vertebra. Under four loading conditions (flexion, extension, and left and right lateral bending), the angle and displacement outputs of the functional spinal units were determined. The results were compared with data obtained from biomechanical experiments in previous studies to validate the model, and we found that our model is effective. In the absence of bone cement in the vertebral body, the force is relatively uniform and small (Fig. 4C). The models with bone cement in the vertebra were composed of two symmetrical spheres (Fig. 4F). In any situation, when the cortical bone is damaged, the force acting on the trabecula is significantly greater than that acting on the intact cortical bone. The dynamic change in maximum von Mises stress on the T12 cancellous bone with stress is shown in Fig. 5A. Moreover, with increased force on the vertebral body, the force on the bone cement and the nearby bone tissue also increases. When the cortical bone is intact, the increase in the mechanical force of the MC-modified PMMA bone cement ranges from 7.51 × 10^5^ to 1.58 × 10^6^ N. When cortical bone defect occurs, the increase in the mechanical force of the MC-modified PMMA bone cement is from 9.58 × 10^5^ to 1.87 × 10^6^ N. When the cortical bone is intact, the increase in the mechanical force of the PMMA bone cement ranges from 1.23 × 10^6^ to 1.68 × 10^6^ N. When cortical bone defect occurs, the increase in the mechanical force of the PMMA bone cement is from 1.07 × 10^6^ to 2.19 × 10^6^ N. When under pressure, the PMMA bone cement and the surrounding bone trabeculae bear more stress, especially at 1000 N. Under 1000 N, the pressure of the PMMA bone cement was 2.19 × 10^6^ N in the Kümmell model and 1.69 × 10^6^ N in the normal model. The pressure of the MC-modified PMMA bone cement was 1.87 × 10^6^ N in the Kümmell model and 1.58 × 10^6^ N in the normal model.

**Figure 6.**
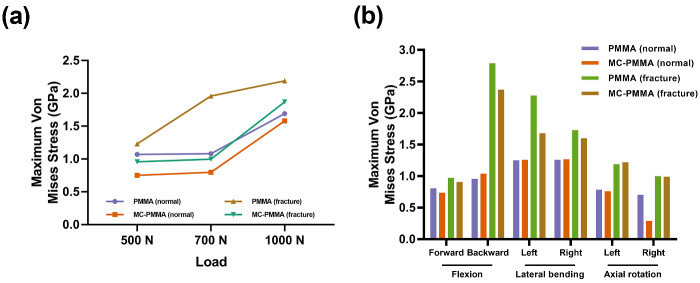
Distribution of vertebral force. (**A**) Maximum von Mises stress of bone cement under 500, 700 and 1000 N of external vertical force. (**B**) Maximum von Mises stress of bone cement under forward and backward flexion, left and right lateral bending, left and right axial rotation

Figure 5B shows a cloud chart of the Kümmell and normal models of human vertebra. For an intact vertebral cortex, the upper and lower surfaces of the bone cement are more stressed (in red) when the vertebral body is subjected to pressure. When the cortical bone of the vertebral body is incomplete, the stress on the area with defect is more obvious (in red). The internal and boundary forces of the PMMA bone cement are greater than those for the MC-modified PMMA bone cement in both models. Moreover, under conditions that simulate normal stress, the loading forces of backward flexion and left/right lateral bending are greater than those of other loading conditions. In the Kümmell model, the maximum pressure of the PMMA bone cement with backward flexion was 2.79 × 10^6^ N and that of the MC-modified PMMA bone cement was 9.58 × 10^5^ N. In the normal model, the maximum pressure of the PMMA bone cement with right axial rotation was 7.03 × 10^5^ N and that of the MC-modified PMMA bone cement was 2.91 × 10^5^ N. The internal and boundary forces of the PMMA bone cement were obviously greater than those of the MC-modified PMMA bone cement in both models, especially with backward flexion and left lateral bending (Fig. 6A and B).

## Discussion

Several treatment options are commonly used for KD, including less invasive interventions (such as nerve block), conservative treatment (such as bed rest and narcotic analgesics) and surgical intervention. Currently, PVP is an effective minimally invasive procedure that provides pain relief and improves spinal instability associated with KD [24].

PMMA bone cement is a widely used filling material in PVP for the treatment of KD in clinic, which is mainly composed of PMMA powder and solvent with injectability, plasticity and self-coagulation [25]. Nevertheless, because of the special pathological changes in KD, in which natural fracture healing is impossible, the interface stability of the bone and bone cement and postoperative spontaneous collapse of the vertebral body could still occur [26]. In addition, with the increasing number of KD cases treated with PMMA bone cement and the prolonged follow-up time, the disadvantages of PMMA bone cement have become increasingly prominent [27]. Thus, modification of PMMA bone cement to improve its clinical effect has received increasing attention. MC has good biocompatibility and osteoinductivity, which is conducive for the formation of new bone. MC-modified PMMA bone cement has low elastic modulus property [28] and good histocompatibility with surrounding bone tissue [14, 29−31]. Therefore, in this study, we performed PVP with MC-modified PMMA bone cement to treat KD to obtain better clinical performance and reduce re-fracture.

The modified bone cement showed good potential in reducing complications of PVP such as re-fracture of the adjacent vertebral body and vertebra height decrease. Because of the relatively high elastic modulus of the PMMA bone cement compared with the natural bone, stress concentration and fractures of adjacent vertebral body could easily occur. With the MC-modified PMMA bone cement, the incidence of adjacent vertebral re-fracture was reduced to 10.00%, which was significantly better than that in the traditional bone cement group (38.46%). Other researches have indicated the strengths and weaknesses of PMMA bone cement in treating KD. Kim *et al*. [32] found that the height of the injured vertebrae decreased by 7.1% at 6 months postoperatively. Zhang *et al*. [33, 34] showed that the vertebral bodies slightly collapsed at the end of the follow-up. For most patients, cement augmentation acts as a supporting block. As PMMA bone cement rarely diffuses into the trabecular bone, the locking effect between the bone and bone cement is insufficient [35]. Given the characteristics of KD, spontaneous fracture of the vertebral body, which could be associated with the obvious force exerted by the bone tissue near the cement, as well as re-compression of the vertebral body would like to occur. Moreover, PMMA bone cement with a high modulus, could break the stress uniformity of the original vertebral body, thereby causing stress concentration in the bone tissue near the bone cement. In the results of finite element analysis, we found that the stress of bone tissue near PMMA bone cement was significantly greater than that of MC-modified PMMA bone cement. When the stress of the bone trabeculae around the cement is substantial, the bone trabeculae are prone to fracture recurrence, which could lead to vertebral collapse. Moreover, MC-modified PMMA bone cement with lower elastic modulus could reduce damage and force to the surrounding bone tissue, thereby preventing spontaneous vertebral collapse in KD in the result of finite element. With the addition of MC, the mechanical properties of the PMMA bone cement have changed significantly and the elastic modulus decreases, which could in turn reduce the hardness of the diseased vertebral body, the interface wear of the bone cement on the diseased vertebral body, and the risk of secondary fracture of the adjacent vertebral body. In summary, the cement forms a stable medium with a certain mechanical strength, which has a supporting role in the vertebral body and is thus beneficial to the recovery of the height of the injured vertebral body [36].

The incorporation of MC into the PMMA bone cement could improve osteointegration and biocompatibility between the host bone tissue and the bone cement. MC in the PMMA bone cement could be replaced by a new bone tissue based on CT findings. In our study, at 6 months and 1 year of follow-up, the BMD of the injured vertebra were significantly higher in the MC-modified PMMA group than in the traditional group, which proved that the modified bone cement has good osteoinductivity and histocompatibility in the vertebra. Such modification of the PMMA bone cement could form a more stable structure with the surrounding bone tissue in the vertebral body, thereby allowing the bone cement to provide a wider support. Thus, the MC-modified PMMA bone cement could reduce slippage, instability and recompression of the vertebra [13].

Adequate cement viscosity is crucial for reducing the risk of PMMA cement leakage [37−40]. Anselmetti *et al*. [41] reported a significant reduction in cement leakage with the use of a high-viscosity cement versus low-viscosity cement, while other studies failed to demonstrate increased risks of leakage using low-viscosity cement [42]. With the addition of MC, the demarcation line between the waiting period and the working period of the MC-modified PMMA bone cement remain to be further clarified; nevertheless, there is a certain viscosity in the waiting period that allows the cement to last until the end of the working period. Despite the ambiguity of the boundaries between the waiting and working periods, we believe that the cement viscosity changes to reach the predetermined area in the vertebral body, achieve a certain degree of dispersion, and form mechanical occlusion and blend with the patients’ autogenous bone.

Regarding pain in patients with KD, pain relief with the MC-modified PMMA bone cement is similar to that with the PMMA bone cement in the short term but is better with the MC-modified PMMA bone cement in the long term. The heat released during the solidification process of bone cement could burn the peripheral nerves around the fracture, thereby relieving the pain due to compression fracture in the short term [43]. The MC-modified PMMA bone cement and PMMA bone cement have the same heat-releasing ability during the solidification process. The long-term effect is mainly associated with the micro-movement of the vertebral fracture. Elimination of the micro-fractures and improvement of vertebral stabilization by the MC-modified PMMA bone cement contributed to the satisfactory clinical outcomes in the long term [44].

## Conclusion

The MC-modified PMMA bone cement could achieve the same analgesic effect as that in with the PMMA bone cement and restore the height of the injured vertebral body after surgery in patients with KD. However, the incidence of re-fracture of adjacent vertebral body significantly reduced and CT values of the injured vertebral body significantly improved after operation with the MC-modified PMMA bone cement. The VAS, ODI, AVH and PVH values of the MC-modified PMMA group were almost the same as those of the PMMA group at the early stage; however, the MC-modified PMMA group has better results at 1-year follow-up. In the finite element analysis, it can be seen that the stress of bone tissue near MC-modified PMMA bone cement is smaller than that of PMMA bone cement, which is more conducive to the uniform stress of vertebral body. Hence, the MC-modified PMMA bone cement appears to have a better clinical performance for the treatment of KD in the long term. Nevertheless, the results of this study are limited by the retrospective design, short follow-up period, small sample size and lack of measurement of cement viscosity.

## Funding

This work was supported by National Key R&D Program of China (No. 2018YFC1105202), the Key research and development plan in Zhejiang province (No. 2020C03041), National Nature Science Fund of China (No. 81871797), Natural Science Fund of Zhejiang Province (LY17H060001). No benefits in any form have been or will be received from a commercial party related directly or indirectly to the subject of this manuscript. We thank Jiye lu, Kai Zhang and other doctors and nurses of the Affiliated Hospital of Medical School of Ningbo University for assisting with the proofreading of this manuscript and the patients who volunteered to take part in the research.

*Conflict of interest statement*. None declared.
